# Effect of LIMA Harvesting Technique on Postoperative Drainage in Off-Pump CABG

**DOI:** 10.5935/1678-9741.20160024

**Published:** 2016

**Authors:** Mehmet Özülkü, Fatih Aygün

**Affiliations:** 1 Başkent University, Konya Research and Medical Center, Turkey.

**Keywords:** Coronary artery disease, Coronary artery bypass, off-pump, Internal mammary-coronary artery anastomosis, Tissue and organ harvesting, Drainage

## Abstract

**Objective:**

We investigated the effects of pedicled and semiskeletonized left internal mammary artery (LIMA) harvesting techniques on postoperative drainage in patients subjected to off-pump CABG, ignoring other advantages or disadvantages of those techniques.

**Methods:**

The present study comprises a total of 160 subjects that underwent coronary artery bypass surgery in our clinic. Data were collected consecutively and retrospectively. An attempt was made to have similar groups in terms of demographic characteristics. Patients that underwent off-pump coronary artery bypass surgery by two surgical teams which differed only in LIMA harvesting technique were dichotomized and compared according to these techniques. The first group (Group 1) consisted of patients in whom LIMA was harvested with surrounding tissues using the pedicled technique. The second group (Group 2) consisted of patients in whom LIMA was harvested using the semiskeletonized technique, with the veins separated from surrounding connective tissues.

**Results:**

The mean amount of drainage in the first 24 hours was 706.1±234.2 ml *vs.* 591±258.8 ml (Group 1 *vs.* Group 2; *P*=0.005), the mean amount of drainage in the second 24 hours was 270±133.6 ml *vs.* 189.4±140.4 ml (Group 1 *vs.* Group 2; *P*<0.001), and the mean amount of total drainage was determined to be 976.1±306.9 ml *vs.* 781.2±335.5 ml (Group 1 *vs.* Group 2; *P*<0.001).

**Conclusion:**

It was observed that semiskeletonized LIMA presents reduced amount of postoperative drainage in the first and second 24-hour periods and total amount of drainage than pedicled LIMA, independent of pleural integrity.

**Table:** 

Abbreviations, acronyms & symbols	
ACT	= Activated coagulation time
BMI	= Body mass index
CABG	= Coronary artery bypass grafting
COPD	= Chronic obstructive pulmonary disease
CVA	= Cerebrovascular accident
CVS	= Cardiovascular surgery
EF	= Ejection fraction
ICU	= Intensive care unit
LIMA	= Left internal mammary artery
PAD	= Peripheral artery disease
TTE	= Transthoracic echocardiography

## INTRODUCTION

Many vascular structures in the body are used as conduits in coronary artery bypass grafting (CABG) surgeries. Left or right internal mammary artery, radial arteries, and great saphenous vein are frequently used as vascular structures. The left internal mammary artery (LIMA) is the most commonly used one because of its long patency, ease of application, and graft-coronary match^[1,2]^.

Mediastinal bleeding and bleeding in the LIMA bed after CABG, as well as bleeding/drainage monitored via the tubes placed in the surgical site, are important complications that could be life threatening. The bleeding itself could be life threatening and the blood products that are to be given may lead to serious complications. Is there a relationship between LIMA harvesting technique and postoperative drainage/bleeding? In general, there are two harvesting techniques. The first is the pedicled LIMA harvesting. In this technique, LIMA is excised together with the surrounding tissues. The LIMA that comprises intrathoracic fascia, veins around the artery, adipose tissue and sometimes muscular tissue is called a pedicled LIMA. The second is the skeletonized/semiskeletonized LIMA harvesting. In this technique, LIMA is separated from the surrounding tissue and only the artery is excised. The LIMA that is excised separately from accompanying veins is called skeletonized whereas LIMA that is excised together with accompanying veins is called semiskeletonized.

The fact that it is within the surgical site and it generally requires opening of the left thoracic space during harvesting have raised some questions. The effect of using LIMA on postoperative drainage and the effect of pleural membrane damage during this procedure on postoperative drainage have been a matter of concern. Studies conducted to find out an answer to these questions demonstrated that drainage is in fact less prevalent during bypass surgeries performed without using LIMA^[3]^. Likewise, it was reported that drainage is less prevalent when pleural integrity has been preserved during LIMA harvesting^[3,6]^.

Arterial spasm and arterial injury can be less common with pedicled harvesting, in which arterial contact is minimum or none. Nevertheless, shorter LIMA is obtained as the tortuous structure has been preserved together with surrounding tissue. The likelihood of arterial spasm and injury is enhanced in the skeletonized or semiskeletonized LIMA harvesting technique. This harvesting technique provides a longer LIMA with less injury to the thoracic wall.

In the present study, effects of pedicled and semiskeletonized LIMA harvesting techniques on postoperative drainage were investigated in patients undergoing off-pump CABG surgery.

## METHODS

### Clinical Characteristics of Patients

The present study comprised a total of 160 subjects who underwent CABG in our clinic. Data were collected consecutively and retrospectively. Approval was obtained from the Ethics Committee. An attempt was made to keep the groups (Group 1, n=65; Group 2, n=95) similar in terms of demographic characteristics.

All patients were questioned about their medical history and underwent detailed physical examination. In the preoperative period, standard preoperative laboratory analyses, pulmonary function test (Spirobank Spirometry, MIR medical International Research Product), transthoracic echocardiography (TTE) (Acuson, Mountain View, Acuson Sequoia C256), and bilateral carotid artery Doppler ultrasonography (Toshiba XARIO prime ultrasound) were performed in the Cardiovascular Surgery (CVS) clinic.

In the preoperative period, clopidogrel (if any) was discontinued 5 days prior to the surgery and acetylsalicylic acid was discontinued one day prior to the surgery in patients with coronary artery stenosis who would undergo off-pump (beatingheart) CABG.

In order to form two homogenous groups, patients who underwent LIMA-Left anterior descending artery CABG were not included in our study. Those were few patients who did not impact the amount of postoperative drainage. We faced surgical hemorrhage in a patient who was examined for postoperative drainage. Therefore, those patients were excluded from our study. Sternal wound infection was not seen in neither group. Postoperative pleural effusions have not been taken into consideration in the study so as not to cause confusion with the evaluation of effusions that occur after the drains are removed.

### Surgical Procedure

Initial isolated CABG surgery was performed in all patients participating in the study. Fentanyl, midazolam and pancuronium bromide were administered for the induction of anesthesia. Standard median sternotomy was performed. LIMA and other vascular conduits (saphenous vein and radial artery) were prepared. Patients who underwent the beating heart technique were heparinized by administering 150 IU/kg heparin sodium. Distal anastomoses were performed using Medtronic Octopus^®^ Evolution Tissue Stabilizer and Medtronic Starfish^®^ Heart Positioners. Whilst the LIMA was used in all cases, the right internal mammary artery was not used. Great saphenous vein and radial artery were used as grafts. Meticulous aseptic technique was implemented in the surgeries. During surgery, the beginnings of ascending aorta and aortic arch were precisely examined by manipulation. The procedure was changed in patients in whom plaque was detected by manipulation, and they were excluded from the study. Unnecessary use of electrocautery was avoided. Hematocrit and hemoglobin values were monitored every 20 minutes after the induction of anesthesia until the end of surgery. Intraoperative blood transfusion was performed when hematocrit values reached 20%. Full vascularization was performed during CABG surgery. Mediastinum and chest drains were placed subxiphoidally. Proximal aortic anastomoses were performed using side clamps. At the end of surgery, an appropriate dose of protamine hydrochloride (Protamin^®^ ampoule 1000 IU/1 ml) was administered to keep the activated coagulation time (ACT) level at 140-150s. Surgical data are shown in Table 1.

**Table 1:** Data according to Groups 1 and 2.

	Group 1 (n=65) (Pedicled LIMA)	Group 2 (n=95) (Semiskeletonized LIMA)	*P* values
Age (mean±SD) (year)	65.3±9.5	63.7±9.1	0.308^T^
Gender (male)	38 (58.5%)	57 (60%)	0.846^P^
Smoking	25 (38.5%)	36 (37.9%)	0.942^P^
COPD	20 (30.8%)	29 (30.5%)	0.974^P^
Hypertension	48 (73.8%)	66 (69.5%)	0.548^P^
PAD	4 (6.2%)	8 (8.4%)	0.763^F^
Preoperative stroke story	6 (9.2%)	2 (2.1%)	0.063^F^
Diabetic nondiabetic	*30 (46.2%)	*59 (62.1%)	*0.046^P^
oral a/d	24 (36.9%)	18 (18.9%)	
parenteral a/d	11 (16.9%)	17 (17.9%)	
Right carotid artery stenosis<%50	22 (33.8%)	27 (28.4%)	*0.356^F^
%50< stenosis<%70	*5 (7.7%)	*5 (5.3%)	
% 70< stenosis<%100	*1 (1.5%)		
stenosis=%100			
Left carotid artery stenosis<%50	23 (35.4%)	33 (34.7%)	*1 F
%50< stenosis<%70	*3 (4.6%)	*7 (7.4%)	
% 70< stenosis<%100	__	__	
stenosis=%100	1 (1.5%)		
BMI (mean±SD)	29.3±4.6	29.1±4.7	0.700^T^
Ejection fraction (mean±SD)	54.7±8.7	52.9±8.9	0.255^T^

T *P* value was presented as a result of Student-t test.

P *P* value was presented as a result of Pearson's qui-square test.

F *P* value was presented as a result of Fisher's Exact test.

* P value was calculated according to carotid artery stenosis <%50.

a/d=antidiabetic agent; BMI=Body Mass Index, SD=Standard deviation; PAD=Peripheral artery disease; COPD=Chronic obstructive pulmonary disease

### LIMA Harvesting Technique

Following the exclusion of left hemithorax through the sternum, the LIMA was explored up to bifurcation distally and up to the subclavian vein branch proximally and removed with the help of electrocautery. Great branches (thicker than 1 mm) were separated from the middle with the help of electrocautery by placing metal arterial clips both in the LIMA and the sternum side. For smaller branches, the clip was placed only in the LIMA side and the sternum side was electrocauterized. In the pedicled harvesting technique, LIMA was excised including thoracic fascia, adipose tissue, veins around LIMA, lymphatic tissue and partial muscular tissue by being separated from the branches 1 cm distance from both sides. In the semiskeletonized harvesting technique, the chest wall was separated from the thoracic fascia so that there was 1 cm left around the LIMA. LIMA then became visible and was separated from its branches with the help of a clip and electrocautery, preserving the veins around it and partially including the adipose tissue. Thoracic muscular tissue was not damaged in any way in the semiskeletonized technique. Arterial and venous tissues, which were made clear of the surrounding adipose tissue as much as possible, were explored and released. In both techniques, electrocautery was used at low voltage. After harvesting, LIMA was wrapped in papaverine-impregnated gauze and thoracic bleeding was checked.

The left pleura was standardly opened either while preparing LIMA during surgery or just after the LIMA had been prepared. Inserting mediastinal and left pleural drains through the subxiphoid area is a standard procedure in our clinic.

### Postoperative Care

After completion of the surgical procedure, patients were admitted to the CVS intensive care unit (ICU). They were monitored in the ICU for hematocrit and hemoglobin at 4-hour intervals. The criterion for blood transfusion in the ICU was a hematocrit value of 28%.

In the postoperative period, 300 mg/day acetylsalicylic acid (Coraspin 300^®^ was commenced together with enteral nutrition. Cefazolin sodium (Cefamezin^®^-IM/IV), which is used as the standard prophylactic antibiotic in our clinic, was given at a dose of 1 g at 30 min before surgery and every 8 hours after surgery for 72 hours. Blood glucose regulation in diabetic patients was strictly provided after surgery with insulin glargine 100 IU/ml (Lantus^®^ flacon) and human soluble regular insulin 100 IU/ml (Humulin-R^®^ flacon). Insulin infusion was not avoided when required. Blood glucose concentration was kept below 200 mg/dl in all diabetic patients.

Patients stayed at ICU for 48 hours and then they were admitted to the CVS clinic in the third 24-hour period after the drains (thoracic and mediastinal drains; they were kept until the drainage became serous and amount of drainage in the last 5 hours was 50 cc) and arterial catheters were removed. Central vascular line was closed on postoperative day 4 in the CVS clinic. The patients were discharged on postoperative days 6 to 11.

### Study Groups

Patients underwent off-pump CABG by two surgical teams and were dichotomized according to two different LIMA harvesting techniques. The first group (Group 1) consisted of patients in whom LIMA was harvested together with surrounding tissues using the pedicled technique. The second group (Group 2) consisted of patients in whom LIMA was harvested with the veins separated from surrounding connective tissues using the semiskeletonized technique.

In order to form a homogenous group, dialysis patients or those with creatinine levels higher than 2 g/dl, patients in whom aortic pathology was detected during surgery and thereby the surgical procedure had to be changed, patients that underwent emergency surgery, patients that underwent redo-CABG, and patients that underwent LIMA-left anterior descending artery (single vascular disease patients) CABG were not included in the study. Moreover, patients that underwent valvular and CABG in the same session, that needed postoperative intra-aortic balloon counterpulsation support, and the cases that were exposed to postoperative exploration for any reason were also excluded to form more homogenous and similar groups.

### Subject Characteristics

Age of all study participants ranged from 27 to 89 years old (mean ± standard deviation 64±4 years old). Of those subjects, 95 (59.4%) were male and 65 (40.6%) were female. There were 114 (71.3%) subjects with hypertension, 61 (38.1%) smokers, and 49 (30.6%) patients with chronic obstructive pulmonary disease (COPD). It was observed that 70 (43.8%) patients had type 2 diabetes and 12 (7.5%) had peripheral artery disease (PAD). Data are shown in Tables 1 to 3, according to groups.

**Table 3:** Postoperative drainage according to Groups 1 and 2.

	Group 1 (n= 65) (Pedicled LIMA)	Group 2 (n=95) (Semiskeletonized LIMA)	*P* values
First 24-hour drainage (ml)	706.1±234.2	591±258.8	0.005^T^
Second 24-hour drainage (ml)	270±133.6	189.4±140.4	<0.001^T^
Total drainage count (ml)	976.1±306.9	781±335.5	<0.001^T^
Erythrocyte suspension (bag)	1.9±1	1.2±1.2	<0.001^T^

T *P* value was presented as a result Student-t test.

### Statistical Analysis

Statistical analyses were done using SPSS 15 (SPSS Inc., Chicago, IL, USA). Statistical significance of nonparametric data comparison between groups was analyzed by Chi-Square Test and Fisher's Exact Test (because observed values were below the expected values). Whilst the parametric data were represented as minimum, maximum and mean ± standard deviation, statistical significance of parametric data between the groups was analyzed by independent Student t-test. The result was considered statistically significant if two-tailed P value was smaller than 0.05 (*P*<0.05).

## RESULTS

### Group Characteristics

In Group 1, male subgroup, the mean age was 63.6±9.1 years old; mean body mass index (BMI) was 28.2±3.4 kg/m^2^ and mean preoperative ejection fraction (EF) was 57.8±7.8%. Average grafts per patient was 2.6±0.7; 34 (89.5%) patients received saphenous vein graft and 10 (26.3%), radial artery graft. In this Group, 4 (10.5%) patients presented history of cerebrovascular accident (CVA) before surgery; 1 (2.6%) patient had right carotid artery stenosis (70%< lesion<100%) and 1 (2.6%), left carotid artery stenosis (70%< lesion<100%). There were 24 (63.2%) smokers, 25 (65.8%) patients with hypertension, 10 (26.3%) with COPD, and 2 (5.3%) with PAD. The mean amount of drainage in the first 24 hours was 735.5±229.5 ml, and in the second 24 hours was 288.1±148.1 ml, with mean total drainage of 1023.6±321.8 ml.

In the Group 1, female subgroup, the mean age was 67.6±9.8 years old; mean BMI was 31±5.4 kg/m^2^ and mean preoperative EF was 50.3±8.1%. The mean number of bypass grafting in CABG was 2.8±0.9; 26 (96.3%) patients received saphenous vein graft and 4 (14.8%), radial artery graft. Two (7.4%) patients had history of CVA before surgery and none had right or left carotid artery stenosis superior to 70%. It was observed that there was 1 (3.7%) smoker, 23 (85.2%) patients with hypertension, 10 (37%) with COPD, and 2 (7.4%) with PAD. The mean amount of drainage in the first 24 hours was 664.8±238.9 ml, in the second 24 hours was 244.4±107.7 ml, with mean total drainage of 909.2±276.6 ml.

In the Group 2, male subgroup, the mean age was 62.6±9.7 years old; mean BMI was 27.7±3.7 kg/m^2^; and mean preoperative EF was 52.5±7.7%. Average grafts per patient were 2.4±1; 49 (86%) patients received saphenous vein graft and 13 (22.8%), radial artery graft. Regarding clinical history, 2 (3.5%) patients had history of CVA before surgery and right or left carotid artery stenosis superior to 70% weren't observed in this group. There were 28 (49.1%) smokers, 38 (66.7%) patients had hypertension, 16 (28.1%) COPD, and 7 (12.3%) PAD. In the first 24 hours the mean amount of drainage 648.2±253.7 ml, and in the second 24 hours, 196.4±142.6 ml, with mean total drainage of 845.6±325.4 ml.

In the Group 2, female subgroup, the mean age was 65.4±7.8 years; mean BMI was 31±5.2 kg/m^2^; and mean preoperative EF was 53.6±10.6. The mean number of grafts per patient was 2.4±1; in 36 (84.7%) patients saphenous vein graft was used and in 9 (23.7%), radial artery. CVA and carotid artery stenosis up to 70% wasn't observed in this subgroup. Medical history of this subgroup included: 8 (21.1%) smokers, 28 (73.7%) patients with hypertension, 13 (34.2%) with COPD, and 1 (2.6%) with PAD. The mean amount of drainage in the first 24 hours was 505.2±245.4 ml, and in the second 24 hours was 178.9±138.3 ml, with mean total drainage of 684.2±331 ml.

**Fig. 1:**
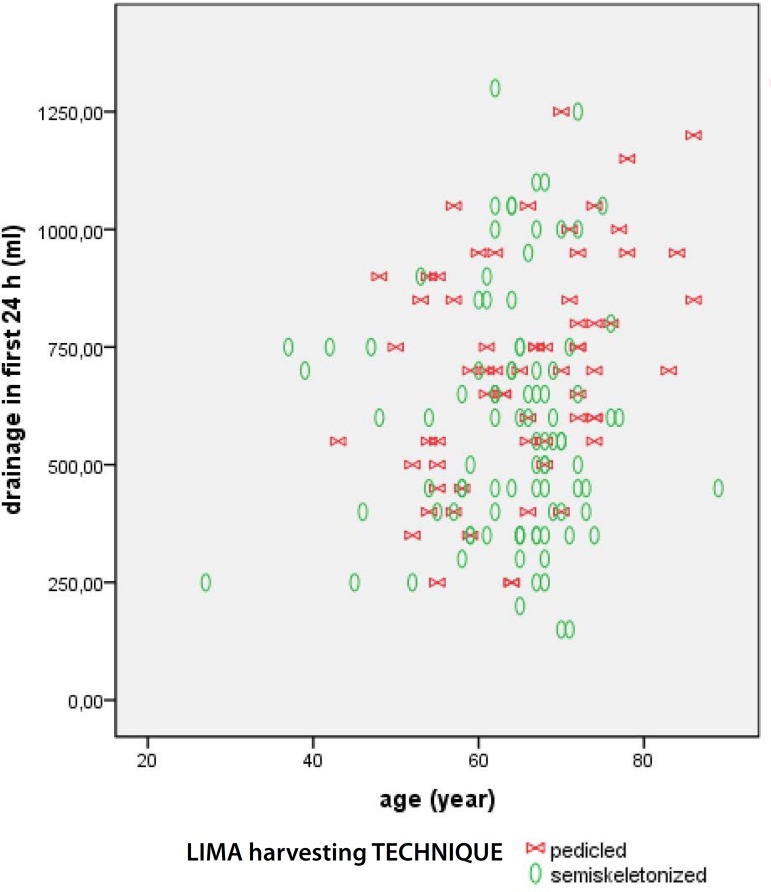
First 24-hour drainage according to age and LIMA harvesting technique.

**Fig. 2:**
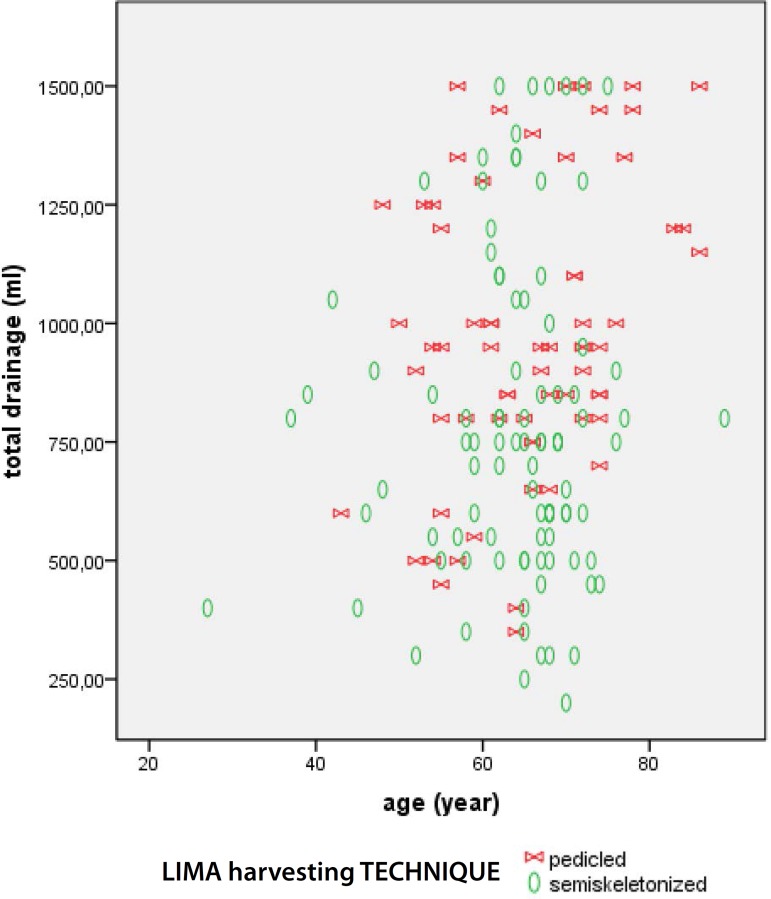
Second 24-hour drainage according to age and LIMA harvesting technique.

**Fig. 3:**
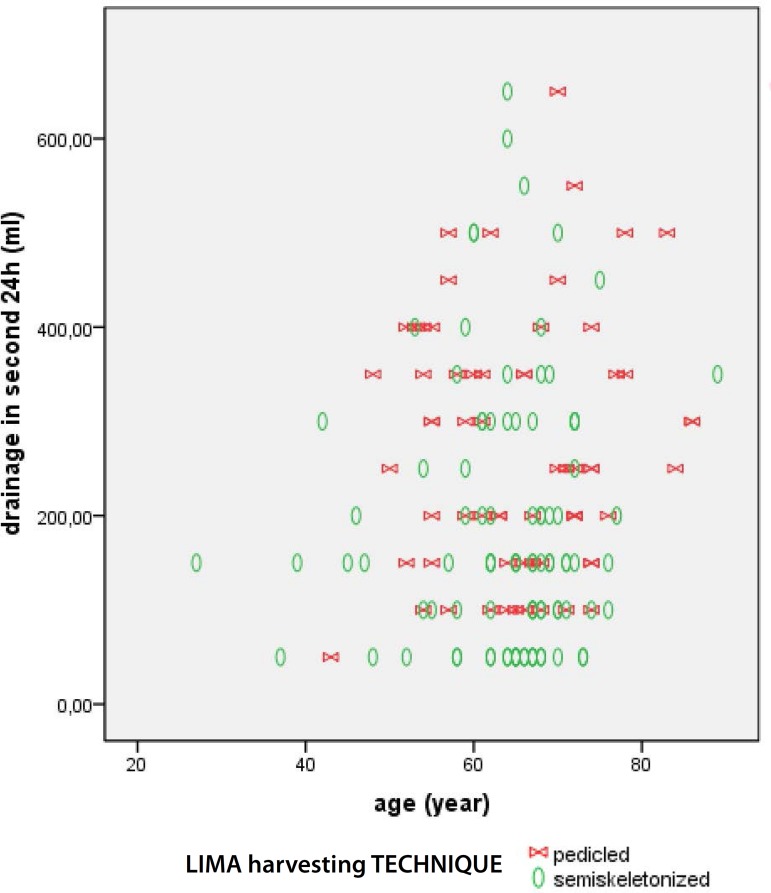
Total drainage according to age and LIMA harvesting technique.

The Figures 1 to 3 present data regarding drainage volume and its association with age.

## DISCUSSION

LIMA is the most commonly preferred arterial graft in CABG surgery due to its long patency, suitability for coronary artery anastomosis, and because it does not require proximal anastomosis^[1,2]^. LIMA harvesting technique has always been a matter of debate for postoperative drainage. In the present study, we retrospectively investigated the effect of LIMA harvesting techniques on drainage in patients who underwent off-pump CABG. We determined that drainage was significantly lower in patients who underwent semiskeletonized LIMA harvesting as compared to patients who underwent pedicled LIMA harvesting (*P*<0.001).

In the wide serial study run by Lamy et al.^[7]^, they compared the early-phase results of patients who were subjected to off-pump and on-pump CABG. They showed that the need for drainage and blood transfusion is less in the group subjected to off-pump CABG. Because there is a difference between the off-pump and on-pump groups in terms of drainage, by taking only off-pump CABG patients we compared the effects of the LIMA harvesting techniques on drainage and proved the superiority of the semiskeletonized group.

It was reported that skeletonized LIMA harvesting supports sternum nutrition by preserving collateral flow and reduces wound site complications^[8]^. There are studies reporting that skeletonized LIMA is longer and provides better free blood flow^[9,10]^. Moreover, it was demonstrated that postoperative chest pain is lower and pulmonary function is better with semiskeletonized LIMA harvesting^[11,12]^.

Kamiya et al.^[13]^ demonstrated that sternal blood flow and oxygen saturation are better with the use of skeletonized LIMA as compared to pedicled LIMA. In their case study, Boodhwani et al.^[11]^ used bilateral internal mammary arteries. The LIMA was harvested as skeletonized whereas the right internal mammary artery was harvested as pedicled. Measuring sternal perfusion of the patients by scintigraphy, they demonstrated higher perfusion on the skeletonized side versus the pedicled side. Sá et al.^[14]^ conducted a meta-analysis by reviewing 22 articles and demonstrated that internal mammary arteries harvesting technique influences the occurrence of sternal wound infection, with lower rate of infection in the skeletonized IMA group. Again, in another study, Sá et al.^[14]^ demonstrated that skeletonized internal mammary arteries harvesting reduces the incidence of mediastinitis. Sá et al.^[15]^ reported that skeletonized LIMA harvesting should be considered in diabetic patients. Sajja^[16]^ determined that cessation of smoking, optimal control of hyperglycemia, sterile operative condition, use of appropriate antibiotics, appropriate internal mammary artery harvesting, and sternal closure reduced mediastinitis.

There may be considerations that better perfusion of LIMA bed could enhance postoperative drainage. Atay et al.^[5]^ suggested that preserved pleural integrity reduces postoperative blood loss independent of LIMA harvesting technique. Göksin et al.^[17]^ dichotomized the patients that underwent pedicled internal mammary artery harvesting according to whether pleural integrity had been preserved or not. They demonstrated lower rates of blood loss and drainage in the group in which pleural integrity had been preserved^[17]^. In the present study, we observed differences in drainage due to harvesting technique though the left pleura had been opened in all patients. Therefore, contrary to the study conducted by Göksin et al.^[17]^, we can say that postoperative blood loss is influenced by the differences in internal mammary artery harvesting technique rather than pleural integrity.

Wimmer-Greinecker et al.^[18]^ reported that sternum pain is lower in CABG surgeries performed using skeletonized internal mammary artery. Boodhwani et al.^[11]^ demonstrated that sternal perfusion is better and sternal pain and tenderness decreases with skeletonized LIMA harvesting. It has been said that surrounding tissues are less damaged in the skeletonized LIMA group. In addition, there are changes during harvesting, including in the pathological process, that are different from the other conduits^[19]^.

Many studies have mentioned benefits of skeletonized LIMA harvesting technique as compared to pedicled LIMA harvesting technique. These benefits include increase in the flow rate of LIMA^[10]^, increase in conduit length^[20]^, decrease in deep sternum infection^[21,22]^, and decrease in postoperative sternal pain. Calafiore et al.^[2]^ investigated patency rate of LIMA in patients who underwent CABG with skeletonized or pedicled internal mammary artery and reported that early and intermediate patency rates were equal.

We have not come across any studies concerning the relationship between postoperative drainage and LIMA harvesting technique. In many recent studies, besides this issue, the connection between intact pleura and postoperative drainage was investigated. According to those studies, keeping pleura intact was the most important factor for decreasing postoperative drainage. However, in our study, the effect of LIMA harvesting technique on postoperative drainage was investigated in all CABG patients whose pleural spaces were opened.

### Study Limitations

In the present study, all patients were Caucasians and therefore do not represent other ethnic groups. Patients with renal insufficiency, dialysis patients or redo CABG cases, among others, who would influence the similarity between the groups, were not included in the study.

## CONCLUSION

In conclusion, it was observed that skeletonized LIMA is more beneficial than pedicled LIMA in terms of amount of drainage in the first 24-hour and second 24-hour postoperative and total amount of drainage, regardless of pleural integrity.

**Table:** 

Authors' roles & responsibilities	
MÖ	Design and drawing of the study; final approval of the
	manuscript
FA	Analysis and/or interpretation of data; statistical analysis;
	writing of the manuscript or critical review of its contents;
	final approval of the manuscript

## Figures and Tables

**Table 2:** Operative data, according to Groups 1 and 2.

	Group 1 (n= 65) (Pedicled LIMA)	Group 2 (n=95) (Semiskeletonized LIMA)	*P* values
Saphenous graft	60 (92.3%)	85 (89.5%)	0.546^P^
Radial artery graft	14 (21.5%)	22 (23.2%)	0.810^P^
LIMA graft	65 (100%)	95 (100%)	1^P^
Numbers of grafting (mean±SD)	2.7±0.8	2.5±0.9	0.136^T^

T *P* value was presented as a result Student-t test.

P *P* value was presented as a result Pearson's qui-square test. LIMA=Left internal mammary artery
